# Screening in Maternity to Ascertain Tuberculosis Status (SMATS) study

**DOI:** 10.1186/s12879-017-2285-0

**Published:** 2017-03-06

**Authors:** Edward Broughton, Samson Haumba, Marianne Calnan, Sandile Ginindsa, Rosanna Jeffries, Gugu Maphalala, Sikhathele Mazibuko, Munamato Mirara, Surbhi Modi, Pasipamire Munyaradzi, Peter Preko, Batsabile Simelane

**Affiliations:** 1 Director of Research and Evaluation, University Research Co., LLC (URC), Bethesda, USA; 2 Chief of Party, URC Swaziland, Mbabane, Swaziland; 3 URC Swaziland, Mbabane, Swaziland; 4 Clinton Health Access Initiative, Dodoma, Tanzania; 5 Swaziland Health Laboratory Services Project, Mbabane, Swaziland; 6 Swaziland National AIDS Program, Mbabane, Swaziland; 7 USAID Swaziland, Mbabane, Swaziland; 8 U.S. Centers for Disease Control and Prevention, Atlanta, USA

**Keywords:** Tuberculosis, Testing, Screening, Diagnosis, Pregnancy, HIV

## Abstract

**Background:**

Diagnosis of tuberculosis is difficult among pregnant women because the signs and symptoms of the disease, such as fatigue, shortness of breath, sweating, cough, and mild fever are similar to some manifestations of pregnancy. It is particularly challenging among HIV-infected women as symptoms are often masked or atypical. Currently, WHO recommends a standard four-symptom screening tool for pregnant and lactating women. There is evidence from South Africa that this screening tool (which, despite complex symptomology in this population, recommends identification of patients with weight loss, fever, current cough and night sweats), may be missing true active TB cases. However there exist several laboratory and clinical procedures that have the potential to improve the sensitivity and specificity of this screening tool.

**Methods:**

This study will evaluate the sensitivity and specificity of the current TB screening tool for pregnant and lactating women, both HIV positive and negative. We will also assess several different enhanced screening algorithm using LAM, IGRA, TST and chest radiography and clinical/laboratory procedures and tests.

The study will use a cross-sectional analytical study design involving pregnant and lactating women up to six months post-delivery attending antenatal or postnatal care, respectively in one of three selected public health units in Swaziland. Participants will be consecutively enrolled and will be in one of four groups of interest: HIV infected pregnant women, non-HIV infected pregnant women, HIV infected lactating women and non-HIV infected lactating women.

**Discussion:**

We expect in conducting all procedures on all participants regardless of result of the symptom screening we may experience a high refusal rate. However, this risk will be mitigated by the long data collection period of five or more months.

**Electronic supplementary material:**

The online version of this article (doi:10.1186/s12879-017-2285-0) contains supplementary material, which is available to authorized users.

## Background

Active tuberculosis (TB) in pregnancy represents a major public health concern globally, given its known adverse effects of maternal and infant mortality and morbidity, such as spontaneous abortion, preterm labor, low birth weight, and increased neonatal mortality [1]. Diagnosis of TB is more difficult among pregnant women because the signs and symptoms of the disease, such as fatigue, shortness of breath, sweating, cough, and mild fever are similar to some manifestations of pregnancy is particularly challenging among HIV-infected women as symptoms are often masked or atypical [2]. Globally, there is need to improve TB screening among both pregnant and lactating women, and to identify algorithms with high case detection to ensure rapid diagnosis and timely treatment initiation [3]. In 2014 the World Health Organization (WHO) advocated for increased research into new diagnostics methods that consider the specific needs of pregnant and lactating women as well as HIV-infected women [4], and affordable TB screening algorithms are urgently needed for this population [2]. Currently, WHO recommends a standard four-symptom screening tool for pregnant and lactating women. However there exist several laboratory and clinical procedures that have the potential to improve the sensitivity and specificity of this screening tool. These include a urine Lipoarabinomannan (LAM) test, Interferon-Gamma Release Assays (IGRA) blood tests, Tuberculin Skin Test (TST) and chest radiography and other medical history.

There is evidence from South Africa that the WHO recommended 4-symptom screen (which, despite complex symptomology in this population, recommends identification of patients with weight loss, fever, current cough and night sweats), may be missing true active TB cases. One South African study published in 2013 found the sensitivity of any one of the four WHO TB symptoms, versus no TB symptoms, was 28% (95% CI 15–46%) among HIV-infected pregnant women [5]. Another study in Kenya found that, compared to mycobacterium TB culture, WHO symptom screening for TB had a sensitivity of 60% [6].

In Swaziland, where the TB incidence is estimated at 1320/100,000 persons in the general population [7], the current TB screening tool is based on the WHO recommended four-symptom screening. Despite high TB occurrence in this setting, including Multi-Drug Resistant (MDR) TB, the sensitivity and specificity of this screening tool has not yet been evaluated in among pregnant and lactating women in Swaziland. This study will evaluate the sensitivity and specificity of the current TB screening tool for pregnant and lactating women, both HIV positive and negative, against the gold standard of Mycobactial growth indicator tube (MGIT) sputum culture. We will also assess several different enhanced screening algorithm using LAM, IGRA, TST and chest radiography and *XpertMTB/RIF (*GeneXpert).

## Methods

The study will use a cross-sectional analytical study design involving pregnant and lactating women attending antenatal or postnatal care, respectively in one of three selected public health units in Swaziland. Inclusion criteria include woman at any stage of pregnancy or lactating woman up to 6 months post- delivery, aged 18 or above, willing and able to provide informed consent, not currently diagnosed as having active TB or TB treatment, not on TB treatment in the past 2 months and not enrolled in any other study less than three months prior. Participants will be consecutively enrolled from these three facilities.

**Fig. 1:**
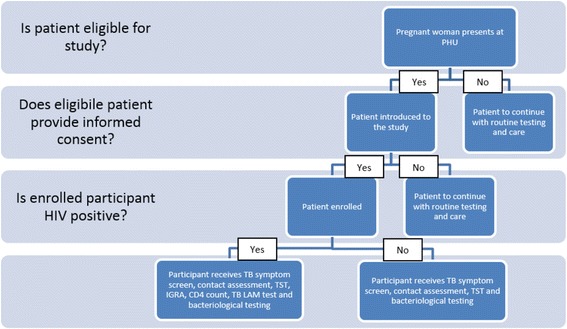
Patient selection criteriaᅟ

There are four groups of interest to this study, namely HIV infected pregnant women, non-HIV infected pregnant women, HIV-infected lactating women and non-HIV infected lactating women (Fig. 1).

Based on detecting differences between important variables of 15% with an alpha of 0.05 and a power of 0.8, the minimum sample size is 183 in each group. In recognition of the need to have a full data for each participant to allow analysis, we will target 250 enrollees per group to allow for missing variables. This allows for comparing factors such as HIV infected pregnant women versus uninfected women.

For each participant enrolled in the study, we will collect clinical and socio-demographic data; results from the WHO four symptom screen; data on whether they were screened for TB at their last appointment; laboratory test results; non-laboratory procedure results and data on participant preferences between TST and IGRA. All variables for the study will be recorded in one single participant data extraction form/questionnaire which will be completed and updated by study nurses (licensed health professionals hired specifically for the study) placed at each study site, and stored in an individual participant file along with the signed informed consent form.

**Fig. 2:**
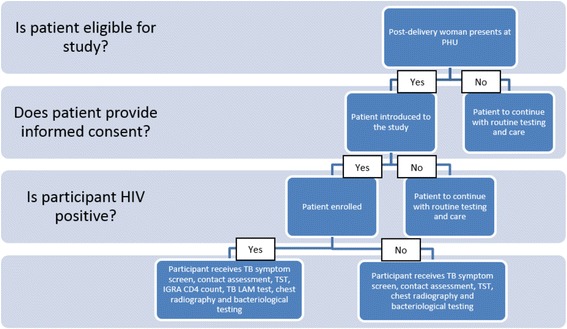
Patient management algorithm

**Fig. 3:**
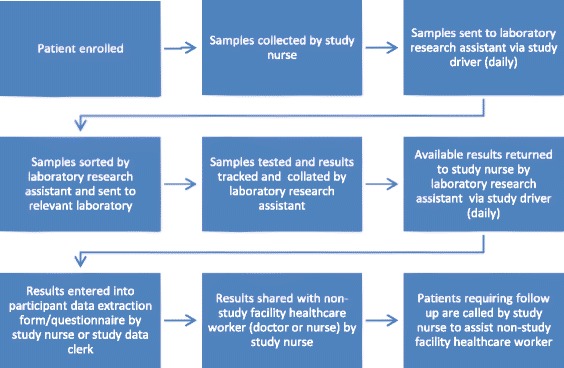
Testing and reporting protocol

In addition to the current four-symptom TB screening (and regardless of its outcome), each participant will undergo all diagnostic tests under investigation in this study. laboratory and non-laboratory procedures, depending on whether they are HIV infected, lactating or pregnant. See Figs. 2 and 3 for proposed study flow diagrams. Conducting these tests and obtaining confirmation of TB status from all participants, both those screening negative and positive on the TB symptom screening tool, will allow identification of the most sensitive screening algorithm, at analysis stage.

Following the giving of informed consent and before collection of samples or undergoing medical procedures, participants will be administered a short questionnaire by the study nurse, which will gather information on the participants’ socio-demographic characteristics including age, marital status, employment status, educational level and living arrangements (living in one room or more). Clinical variables will also be collected in this tool including HIV-status, ART-status, stage of pregnancy or post-delivery stage and whether or not the participant was BCG vaccinated. The questionnaire will additionally collect information on the presence and duration of the four TB symptoms of weight-loss, fever, persistent cough, night sweats.

The study nurses will be responsible for obtaining the necessary biological samples from each participant. These include two sputum samples (one for GeneXpert and one for smear microscopy and culturing); one urine sample (from HIV-infected participants) for TB LAM; and one blood sample for IGRAs. If the patient is HIV-infected, a blood sample will be taken for IGRAs and an additional blood sample will be collected for same day CD4-count testing, given that CD4 counts fluctuate and that the most recent count may not be valid. Sample collection will be according to national standard operating procedures (SOPs) for blood and urine collection, and taking into consideration Urine LAM and IGRA test manufacturer instructions.

Study nurses will be trained on supervised coughing, however the sputum collection may present a challenge given that many participants may not have an expectorant cough. To help overcome this challenge, nebulizer kits will be provided to each site for sputum induction purposes for those unable to cough out spontaneously. The samples will be visually checked by the study nurses before accepting. If a participant either refuses to make use of the nebulizer of if the nebulizing fails to collect the sample, participants will be given sputum bottles and given the opportunity to bring an early morning sputum sample at the next appointment (for TST check-up) or within a maximum of two weeks.

All samples will be stored in accordance with their respective storage SOP and sent to the nearest referral laboratory for testing. All laboratories are certified by the Swaziland MOH and have quality assurance measures in place. Note that the IGRA test is not currently conducted in the national government laboratories so blood samples for these will be sent to a private laboratory that does offer the testing service (Lancet Laboratories) which is nationally accredited. The CD4 cell count tests will also be conducted by the Lancet laboratories to prevent additional burden on the government laboratory and to mitigate the risk of reagent stock-outs. The sections below provide additional details on the tests to be done. The National TB Reference Laboratory was used for sputum cultures. It participates in an external quality assessment (EQA) scheme with the Supra National Laboratories in Uganda and Antwerp Laboratory in Belgium. It is currently enrolled in Strengthening Laboratory Management through Accreditation (SLMTA) program and is in the process of acquiring Southern African Development Community Accreditation Service (SADCAS) accreditation.

### Analysis

Sensitivity, specificity, Area under the Receiver Operating Characteristic (AUROC), positive predictive values and negative predictive values of the current four-symptom screening tool will be calculated against MTB culture results, the ‘gold standard’ for TB diagnosis (Table 1).

**Table 1:** HIV-positive TB diagnostics results

		Sputum culture		
		Pos	Neg	
Test algorithm	Pos	3	20	23
	Neg	1	176	181
		4	196	200

Sensitivity = 0.75

Specificity = 0.90 with ability to detect a 7% difference between alternate algorithms (*p* < 0.05)

A series of “what if” analyses will be conducted for each of the observations collected in the study with the different possible scenarios as the screening and testing algorithms. Additional file 1 below provides an illustration of these hypothetical algorithms (Table 2). For the algorithm including LAM, the analysis will stratify the data by CD4 count levels. Using participants’ data collected from the TB screening and laboratory tests, the sensitivity, specificity positive predictive values and negative predictive values of each algorithm can be calculated. However, clinical decisions on patient management will not include TB LAM until when it is incorporated in the guidelines.

**Table 2:** Laboratory test result options

Test	Result options
TB LAM	Positive; Negative; Missing Result
CD4 count	Whole number; Missing Result
Xpert MTB/RIF	MTB Detected; MTB Not detected; Missing Result
Xpert MTB/RIF resistance	If MTB Detected, specify RIF Resistant; RIF Susceptible; RIF Indeterminate
Smear Microscopy	AFB positive; AFB negative; Missing Result
Liquid Culture	MTB Positive; MTB Negative; Contaminated specimen; Missing Result
DST	If MTB Culture Positive, list resistance patterns to first line drugs
IGRA	Positive; Negative; Indeterminate; Missing Result

### Trial status

Data collection is currently underway in the three participating facilities. It is anticipated that it will be completed in the first half of 2016. Data collection and write up will proceed in the six months following.

## Discussion

One issue we expect in conducting all procedures on all participants regardless of result of the symptom screening is that we may experience a high refusal rate. This is in part due to the time required to participate (approximately 60 min), as well as required follow-up appointment for TST test skin reading. However, we expect that this risk will be mitigated by the long data collection period (six months). We also recognize that it is possible that despite the training of those collecting the samples and the careful quality controls at the laboratories analyzing the samples, not all samples will return definitive results for all tests. To address this limitation, we are aiming for a sample size 25% larger than the minimum required sample size.

Enrolling the first six eligible patients on a first-come, first-served basis may unknowingly introduce selection bias. Anecdotal evidence suggests that most patients present at the facility in the morning, hence the need to introduce selection criteria. Given that there is no reason to believe that those patients arriving first will differ biologically or otherwise from those arriving after the selection of the first six, we anticipate that this selection process will not bias the study’s results.

There may be limitations in the laboratory tests themselves. In particular, we note that the evidence base for IGRAs and TST is inconclusive and the former is not currently recommended by the WHO. Likewise, the use of urine LAM test for screening in this population group has not been thoroughly evaluated. These limitations are accounted for in the study design, by conducting these novel tests in conjunction with routine tests for bacteriological confirmation. Only bacteriological confirmation through WHO Recommended Diagnostics (Xpert MTB/RIF, smear microscopy or MTB sputum culture) or clinical diagnosis will be used to initiate a participant on TB treatment, and this decision is at the discretion of the routine clinician/nurse. As these have not been recognized by the WHO, doctors will be blinded from results of the IGRA and the TB LAM tests to ensure that these do not influence clinical decisions. TST will be used as the diagnostic test for latent TB and to inform the decisions on whether to initiate a patient on Isoniazid Preventive Therapy (IPT).

### Research to policy

This study aims to contribute to the evidence used by policymakers in defining the national standard for TB detection in this population in Swaziland. It is the intent that this will inform development of the treatment cascade from testing and diagnosis to treatment and treatment completion and cure.

## Additional file

Additional file 1: Figure S1. Hypothetical screening and diagnostic algorithms for pregnant and lactating women. (DOCX 43 kb)

## Abbreviations

ART: Anti-retroviral therapy
AUROC: Area under the Receiver Operating Characteristic
BCG: Bacillus Calmette-Guerin
CDC: Centers for Disease Control
HIV: Human immunodeficiency virus
IGRA: Interferon-Gamma Release Assays
IPT: Isoniazid Preventive Therapy
LAM: Lipoarabinomannan
MDR-TB: Multi-drug resistant tuberculosis
MTB: Mycobacterium tuberculosis
RIF: Rifampin
SOP: Standard operating procedure
TB: Tuberculosis
TST: Tuberculin Skin Test
WHO: World Health Organization
