# Supplementary material for: Screening in Maternity to Ascertain Tuberculosis Status (SMATS) study
Source: BMC Infect Dis. 2017 Mar 6;17:191. doi: 10.1186/s12879-017-2285-0 (PMC5340038; doi:10.1186/s12879-017-2285-0)

**Additional file**

Figure S1: Hypothetical screening and diagnostic algorithms for pregnant and lactating women

1. For all pregnant and lactating women, HIV+ and HIV-


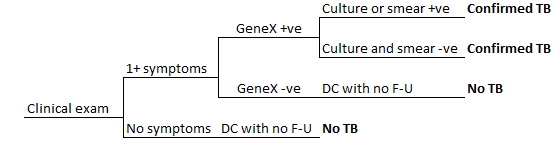


1. For all pregnant and lactating women, HIV+ and HIV-


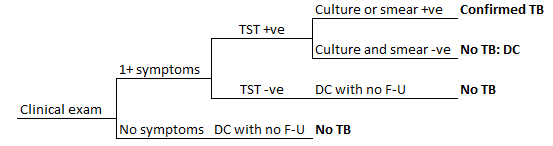


1. For all pregnant and lactating women, HIV+ and HIV-


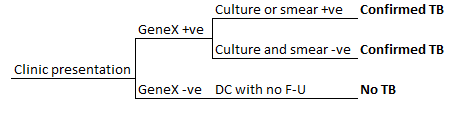


1. For all pregnant and lactating women, HIV+ and HIV-


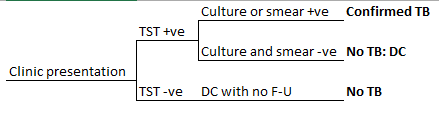


e. For all pregnant and lactating women, HIV+ and HIV-


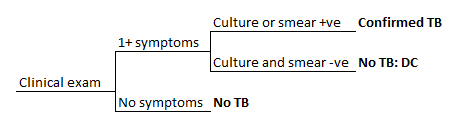

Supplement: Additional file 1: Figure S1. — Hypothetical screening and diagnostic algorithms for pregnant and lactating women. (DOCX 43 kb) [file 12879_2017_2285_MOESM1_ESM.docx]
